# Successful Establishment of Somatic Embryogenesis and Shoot Organogenesis Systems in *Catalpa bungei* C.A.Mey

**DOI:** 10.3390/plants14172688

**Published:** 2025-08-28

**Authors:** Jingshuang Sun, Jiewen Li, Mengnan Zhao, Guangshun Zheng, Jing Zhang, Bao Di, Wenjun Ma, Junhui Wang, Ruiyang Hu

**Affiliations:** 1 State Key Laboratory of Tree Genetics and Breeding, Experimental Center of Forestry in North China, Chinese Academy of Forestry, Beijing 100091, China; sjshuang1129@163.com (J.S.); lijiewen@caf.ac.cn (J.L.); guangshunzheng@163.com (G.Z.); 2 National Forest Germplasm Resource Bank of Representative Plants in North China, Chinese Academy of Forestry, Beijing 102300, China; 3 Horticultural College, Hebei Agricultural University, Baoding 071001, China; zhaomengnan526@163.com (M.Z.); zzj09042023@163.com (J.Z.); dibao666@126.com (B.D.); 4 Key Laboratory of Tree Breeding and Cultivation of State Forestry Administration, Research Institute of Forestry, Chinese Academy of Forestry, Beijing 100091, China; mwjlx.163@163.com (W.M.); wangjh@caf.ac.cn (J.W.)

**Keywords:** *Catalpa bungei*, regeneration, somatic embryogenesis, shoot organogenesis, histology, embryogenic callus

## Abstract

*Catalpa bungei* C.A.Mey is an economically significant deciduous tree valued for timber production and landscaping applications. An efficient regeneration system is crucial for clonal propagation and serves as a foundation for future molecular breeding in *C. bungei*. This study established two in vitro regeneration pathways—indirect somatic embryogenesis and shoot organogenesis utilizing mature zygotic embryos as explants. Primary callus was induced from cotyledon, hypocotyl, and plumule explants. A high frequency (45.73%) of yellow-green compact callus was achieved on De-Klerk and Walton (DKW) medium supplemented with 2.0 mg/L 6-BA, 1.0 mg/L zeatin (ZT), and 0.1 mg/L NAA. Subsequent transfer to 1.5× Murashige and Skoog (MS) medium containing 1.5 mg/L 6-BA, 0.2 mg/L ZT, and 0.1 mg/L NAA yielded the highest embryogenic callus induction rate (16.67%). Embryogenic callus demonstrated bipotent potential, generating both adventitious shoots and somatic embryos under specific hormonal conditions. Histological analyses confirmed the typical developmental stages of somatic embryos, from globular to cotyledonary forms, validating the embryogenic origin of regenerated structures. Furthermore, hormone or osmotic additives such as abscisic acid (ABA), Phytagel, and polyethylene glycol 4000 (PEG4000) significantly enhanced somatic embryo induction, with Phytagel at 5.0 g/L achieving the highest rate (76.31%). For shoot organogenesis, the optimal hormonal combination of the 0.6 mg/L 6-BA, 0.4 mg/L KT, and 0.15 mg/L NAA achieved the highest bud induction rate (88.89%) and produced an average of 4.07 adventitious buds per explant. This study presents an efficient regeneration system for *C. bungei*, providing a practical platform for large-scale propagation and basis for biotechnological applications in woody plants.

## 1. Introduction

*Catalpa bungei* C.A.Mey, a member of the Bignoniaceae family [1], is a fast-growing, deciduous tree species native to China. The species is highly regarded for its ornamental characteristics, high-quality timber, and adaptability to various environmental conditions. Consequently, it has been extensively utilized in urban greening, landscaping, and ecological restoration initiatives [2,3]. Beyond its esthetic and ecological functions, *C. bungei* demonstrates significant potential as a genetic resource for timber improvement and sustainable wood production [4,5]. However, traditional propagation methods, including seed sowing and stem cuttings, are constrained by seed dormancy, low germination rates, and poor rooting efficiency, thus restricting large-scale cultivation and genetic improvement efforts [6].

In vitro regeneration systems serve as essential tools for clonal propagation, germplasm preservation, and transgenic research. Among these methods, somatic embryogenesis and shoot organogenesis represent two fundamental regeneration pathways successfully implemented in numerous woody plants [7,8,9,10]. Somatic embryogenesis facilitates the formation of bipolar structures analogous to zygotic embryos, enabling large-scale clonal propagation [7,8,9]. Shoot organogenesis promotes the direct induction of unipolar adventitious shoots, offering an expedited route to whole-plant regeneration [10,11]. Developing an effective dual-regeneration protocol in *C. bungei* would substantially enhance its potential in tree breeding, mass propagation, and biotechnological applications.

The efficacy of shoot embryogenesis and somatic embryogenesis primarily relies on the successful induction and proliferation of embryogenic callus, which is significantly influenced by plant growth regulators (PGRs) [12,13,14]. The ratio, type, and concentration of these PGRs determine the morphogenic pathway and developmental trajectory of in vitro cultures [13,15,16]. The balance and concentration of these hormones govern the fate of cultured cells, affecting callus formation, embryogenic competence, and shoot induction. Auxins such as 2,4-dichlorophenoxyacetic acid (2,4-D), α-naphthaleneacetic acid (NAA), and indole-3-butyric acid (IBA) are frequently employed to promote callus initiation and embryogenic transition [17,18,19], while cytokinins including 6-benzylaminopurine (6-BA) and zeatin (ZT) are essential for maintaining embryogenic potential and inducing shoot regeneration [20,21]. Thidiazuron (TDZ), a potent cytokinin-like compound, has demonstrated effectiveness in inducing organogenesis in woody species [22,23].

Additionally, basal medium composition—including macronutrient and micronutrient concentrations—significantly influences cell differentiation, callus morphology, and embryo development. Media such as Murashige and Skoog (MS), half-strength MS (1/2 MS), and De-Klerk and Walton (DKW) have demonstrated effectiveness in somatic embryogenesis induction in species like *Jatropha curcas* Linn., *Coffea arabica*, *Eucalyptus* spp., and *Rosa hybrida* [24,25,26,27]. Furthermore, abscisic acid (ABA) and osmotic stressors, such as polyethylene glycol 4000 (PEG4000), and Phytagel, have been reported to improve somatic embryo maturation and stress responsiveness in conifers and *Theobroma cacao* [7,24,28].

Although prior studies on *C. bungei* have established the feasibility of inducing embryogenic callus from immature or mature zygotic embryos, stem segments, or leaves [15,29,30], these systems typically require extended culture durations and demonstrate low efficiency. Additionally, genotype dependence, inconsistent responses, and limited understanding of stage-specific requirements continue to affect reproducibility. Despite gradual progress, significant need persists for systematic optimization of PGR combinations, basal media, and stress-related additives across distinct regeneration stages, including primary callus formation, embryogenic callus maintenance, somatic embryogenesis maturation, and shoot embryogenesis induction.

In this study, we developed a comprehensive and efficient dual-regeneration system for *C. bungei* genotype 12–13 through both somatic embryogenesis and shoot organogenesis pathways. To enhance understanding of the somatic embryogenesis pathway, we conducted histological analyses to characterize the cellular origin and developmental progression of somatic embryos. A systematic evaluation of basal media (e.g., MS and DKW) and diverse combinations of PGRs (2,4-D, NAA, 6-BA, ZT, TDZ, IBA) was performed to assess their effects on callus induction, embryogenic callus proliferation, and the regeneration of adventitious shoots. Additionally, we examined the impact of hormone and osmotic additives—ABA, PEG4000, and Phytagel—on somatic embryogenesis induction. These results provide mechanistic insights into regeneration plasticity in *C. bungei* and establish foundation for future applications in clonal propagation, genetic transformation, and molecular breeding.

## 2. Results

### 2.1. Establishment and Histological Characterization of Somatic Embryogenesis in C. bungei

**Figure 1:**
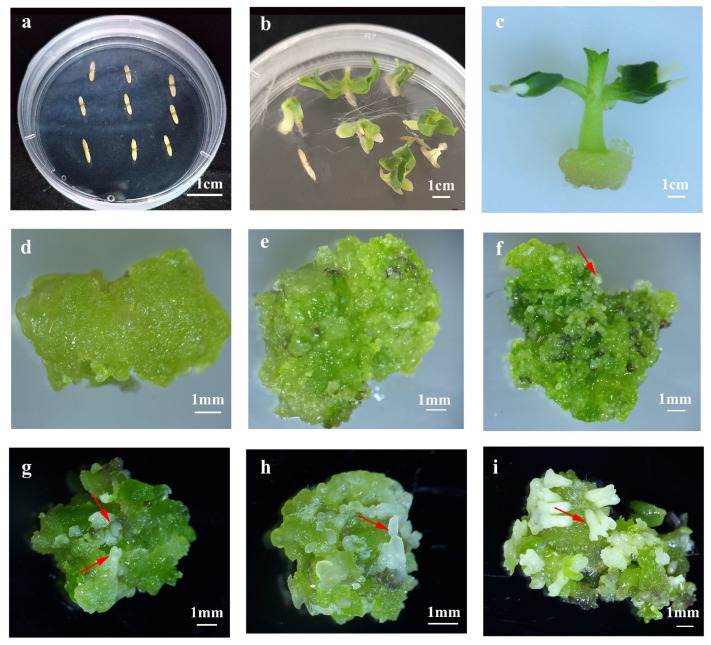
**Morphological progression of somatic embryogenesis induced from mature zygotic embryos (ZEs) of *C. bungei* (genotype 12–13) during in vitro culture.** ZEs were cultured on callus induction medium (CIM, MS-based) and subsequent embryogenic callus/maintenance media with 3 biological replicates per stage. (**a**,**b**) Early morphogenic responses of ZEs after 14 days on CIM: cotyledon expansion and hypocotyl swelling. (**c**) Compact green callus developed at wound sites of isolated hypocotyl explants after 20 days on CIM. (**d**) Yellow-green and compact callus (YGC) with embryogenic potential formed after subculture of green callus. (**e**) Protuberances emerging on YGC during continued subculture. (**f**) Globular somatic embryo (red arrow indicates globular somatic embryo). (**g**) Heart-shaped somatic embryo (red arrow indicates heart-shaped somatic embryo). (**h**) Torpedo-shaped somatic embryo (red arrow indicates torpedo-shaped somatic embryo). (**i**) Cotyledonary somatic embryo (red arrow indicates cotyledonary somatic embryo). All scale bars = 1 mm.

To establish a robust regeneration system in *C. bungei*, we initially investigated whether mature zygotic embryos (ZEs) could initiate somatic embryos through in vitro culture using genotype 12–13. After 14 days on callus induction medium, ZEs exhibited early morphogenic responses including cotyledon expansion and hypocotyl swelling (Figure 1a,b). When cotyledon, hypocotyl, and plumule were cultured separately, compact green calli developed at wound sites after 20 days (Figure 1c). Upon subculture, these calli gradually transformed to yellow-green and compact callus (YGC), a key intermediate with embryogenic potential (Figure 1d). Continued subculture resulted in the emergence of protuberances (Figure 1e), followed by typical embryogenic stages—globular (Figure 1f), heart-shaped (Figure 1g), torpedo-shaped (Figure 1h), and cotyledonary somatic embryos (Figure 1i). These morphological transitions confirmed successful somatic embryogenesis induction in *C. bungei* from multiple ZE-derived explants.

**Figure 2:**
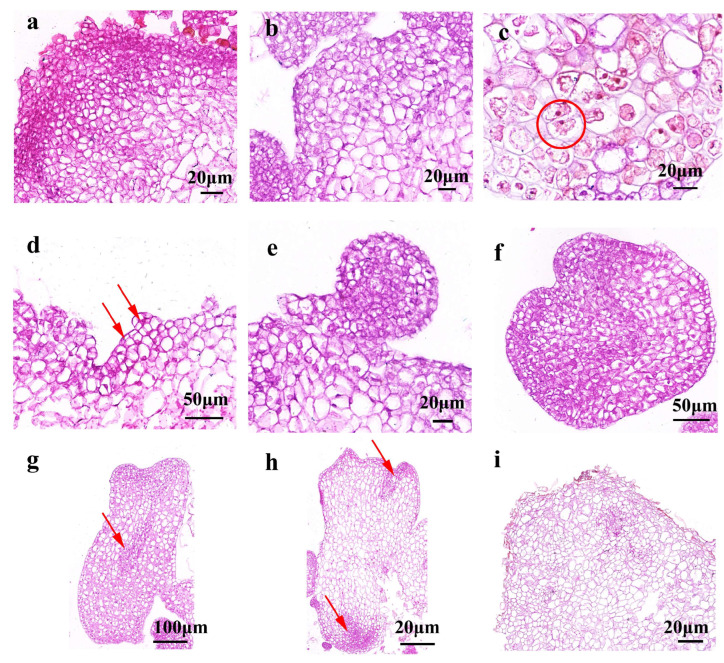
**Histological characterization of indirect somatic embryogenesis in *C. bungei* (genotype 12–13).** Samples were collected at key somatic embryogenesis stages, fixed in FAA solution for 1 week, then dehydrated in graded ethanol, cleared with xylene, and embedded in paraffin. 8–10 µm thick sections were cut with a LEICA RM2245 microtome, stained with hematoxylin, and visualized under a LEICA ICC50 HD light microscope. (**a**,**b**) Early-stage embryogenic cells from epidermal/subepidermal cells, showing dense cytoplasm, thickened cell walls, and prominent nuclei (scale bar = 20 μm). (**c**) Paired pro-embryogenic cells formed by anticlinal division (red circle; scale bar = 20 μm). (**d**) Early pro-embryo with 4–8 cell clusters (from symmetric divisions, red arrows; scale bar = 50 μm). (**e**) Globular-stage somatic embryo (scale bar = 20 μm). (**f**) Heart-shaped somatic embryo (scale bar = 50 μm). (**g**) Torpedo-stage somatic embryo with initial vascular differentiation (red arrow; scale bar = 100 μm). (**h**) Cotyledonary somatic embryo with clear cotyledons, shoot apical meristem, and root apical meristem (red arrows; scale bar = 20 μm). (**i**) Non-embryogenic callus with large vacuolated cells, disorganized structure, and no meristematic zones (scale bar = 20 μm).

To confirm the embryogenic identity and developmental origin of these structures, histological analysis was conducted (Figure 2). Early-stage embryogenic cells originated from single epidermal or subepidermal cells and exhibited dense cytoplasm, thickened cell walls, and prominent nuclei (Figure 2a,b). These cells underwent anticlinal division to form paired pro-embryogenic cells (Figure 2c, red circle), followed by symmetric divisions to generate 4–8 cell clusters typical of early pro-embryo formation (Figure 2d, red arrows). These clusters advanced through the globular (Figure 2e), heart-shaped (Figure 2f), and torpedo-shaped stages (Figure 2g, red arrow), with initial vascular differentiation observed at the torpedo stage. At the cotyledonary stage, embryos displayed well-defined cotyledons, shoot and root apical meristems, and hypocotyls (Figure 2h, red arrows). In contrast, non-embryogenic calli showed large vacuolated cells with disorganized structure and lacked meristematic zones (Figure 2i).

Collectively, these results demonstrate the successful establishment of indirect somatic embryogenesis in *C. bungei*, initiated from ZE-derived explants. The process exhibits distinct morphological and histological features similar to zygotic embryogenesis, indicating its potential for efficient plant regeneration.

### 2.2. Establishment of Shoot Organogenesis in C. bungei

**Figure 3:**
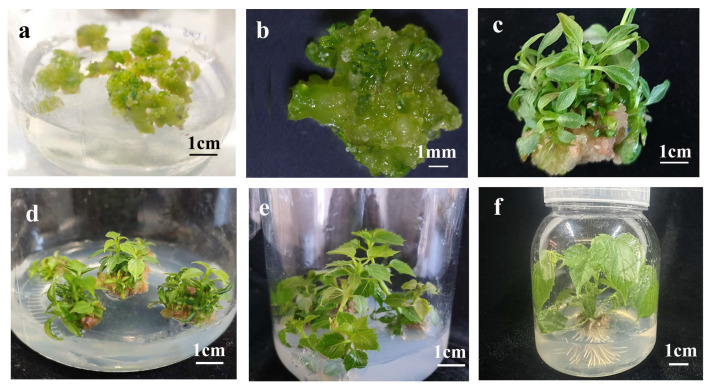
**Morphological progression of indirect shoot organogenesis from embryogenic callus of *C. bungei* (genotype 12–13).** (**a**) Adventitious bud protrusions emerging from embryogenic callus after 30 days of culture on shoot organogenesis medium (SOM) (scale bar = 1 cm). (**b**) Higher-magnification view of adventitious bud initiation (scale bar = 1 mm). (**c**,**d**) Clusters of adventitious shoots with well-formed leaves and petioles developed from the callus (scale bars = 1 cm for both). (**e**) Elongated adventitious shoot after individual subculture on fresh SOM (scale bar = 1 cm). (**f**) Adventitious shoot successfully rooted within 20 days after transfer to rooting medium (scale bar = 1 cm).

Based on the embryogenic competence of YGC, we investigated its potential for shoot organogenesis under inductive conditions. Embryogenic callus tissues were transferred to shoot organogenesis medium (SOM). After 30 days, adventitious bud protrusions emerged from the callus surface (Figure 3a,b) and developed into clusters of adventitious shoots with well-formed leaves and petioles (Figure 2c,d). When isolated and subcultured individually, these shoots exhibited further elongation (Figure 3e), and successfully rooted within 20 days after transfer to rooting medium (Figure 3f).

These findings demonstrate the successful establishment of shoot organogenesis in *C. bungei*, indicating the pluripotent nature of YGC. The callus demonstrates the capacity for both somatic embryogenesis and shoot organogenesis, depending on the hormonal and cultural conditions. This dual regenerative capability provides two efficient and complementary in vitro regeneration pathways for *C. bungei*, enabling flexibility for applications such as vegetative reproduction and genetic transformation.

### 2.3. Effect of Plant Growth Regulators and Explants on Primary Callus Induction

To establish of both somatic embryogenesis and shoot organogenesis in *C. bungei*, primary callus induction was investigated using mature ZE-derived explants, including cotyledons, hypocotyls, and plumules. The formation of YGC essential for both somatic embryogenesis and shoot organogenesis, served as a critical indicator. The study systematically evaluated the effects of explant type and PGRs on primary callus quality and YGC induction.

**Figure 4:**
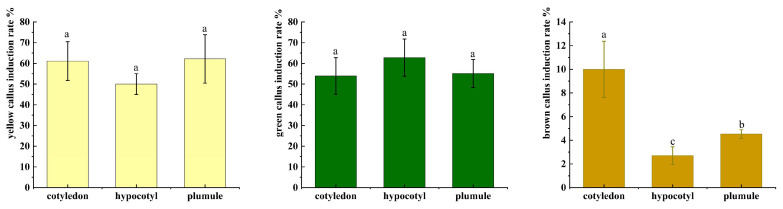
**The effect of different explants (cotyledons, hypocotyls, and plumules) derived from mature zygotic embryos on the induction rate of green callus in *C. bungei* (genotype 12–13).** Different letters in each bar show significant differences by Duncan’s multiple range tests (*p* ≤ 0.05).

Initial evaluation focused on YGC induction rates among different explant types: cotyledons, hypocotyls, and plumules (Figure 4). All explants demonstrated the ability to generate YGC, with no statistically significant differences observed among them (*p* > 0.05). However, hypocotyls displayed the highest mean induction rate, indicating enhanced responsiveness to callus induction conditions.

**Figure 5:**
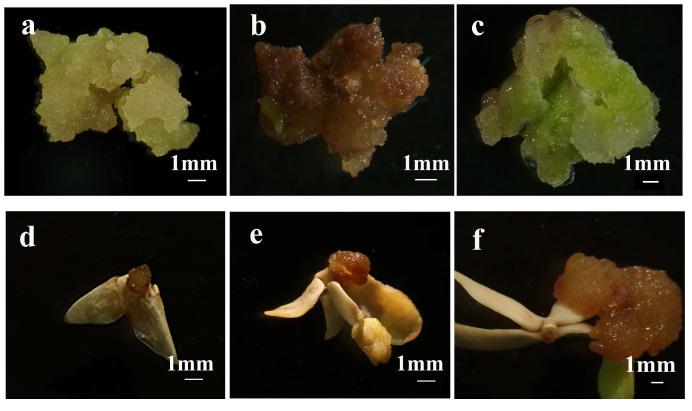
**Morphological characteristics of different callus types induced from mature zygotic embryos of *C. bungei* (genotype 12–13) on callus induction medium (CIM, MS-based).** Calli were cultured for 20 days under controlled conditions (25 ± 2 °C, 16 h light/8 h dark photoperiod, light intensity 40 μmol·m^−2^·s^−1^), with 3 biological replicates per treatment. (**a**) Yellow callus, (**b**) brown callus, and (**c**) green callus induced on CIM with 6-BA and NAA. (**d**–**f**) Brown calli formed on CIM containing 2,4-D, exhibiting necrosis during prolonged subculture. All scale bars = 1 mm.

Subsequently, the effects of PGR combinations on primary callus morphology and frequency were assessed. Mature zygotic embryo-derived explants (cotyledons, hypocotyls, and plumules) were cultured on callus induction medium (CIM) with varying PGR concentrations. After 20 days, three morphologically distinct callus types emerged: green, yellow and brown (Figure 5a–c). Among the tested media, CIM4 (2.0 mg/L 6-BA and 0.2 mg/L NAA) produced the highest induction rate of green callus (58.30%), significantly exceeding medium with lower 6-BA concentration (CIM1-CIM2, *p* ≤ 0.05) (Table 1). Conversely, the addition of 2,4-dichlorophenoxyacetic acid (2,4-D) in CIM7–CIM9 predominantly resulted in brown callus formation with minimal green callus (Table 1), and these brown tissues exhibited necrosis during prolonged subculture (Figure 5d–f).

In summary, these findings indicate that the combination of 6-BA and NAA, without 2,4-D, is more efficient for inducing green and compact callus suitable for further somatic embryogenesis or shoot organogenesis.

### 2.4. Effect of Basal Media and Plant Growth Regulators on Yellow-Green Compact Callus Induction During Subculture

Following the identification of factors affecting primary callus induction, the study examined factors influencing YGC formation during subculture. This specific callus type previously demonstrated development into embryogenic callus in subsequent stages (Figure 1d,e), establishing it as a crucial intermediate for successful somatic embryogenesis. The effects of different basal media and PGR concentrations on YGC proliferation during subculture were systematically evaluated (Table 2).

Among the three basal medium tested, DKW medium supported the highest YGC induction rate, significantly exceeding rates observed on Woody Plant Medium (WPM) and MS medium (*p* ≤ 0.05). Furthermore, within the DKW medium, increased cytokinin and auxin concentration, particularly 2.0 mg/L 6-BA, 1.0 mg/L ZT and 0.1 mg/L NAA, substantially improved callus quality and frequency (45.73% in subculture medium 5: SCM5). In contrast, given the same concentration of cytokinin, lower NAA concentration (0.01 mg/L in SCM6) significantly reduced YGC induction to 7.69%, emphasizing the essential role of auxin balance during callus induction of *C. bungei*.

### 2.5. Effects of Basal Medium and Plant Growth Regulator Combinations for Inducing Embryogenic Callus from Yellow-Green Compact Callus

To facilitate subsequent somatic embryogenesis, the study assessed whether YGC derived from the optimized subculture conditions could be induced to form embryogenic callus under different combinations of basal media and PGRs.

Embryogenic callus formation occurred exclusively on MS and 1.5 × MS media, with no formation observed on DKW medium across all hormone combinations tested (Table 3). The highest embryogenic callus induction rate (16.67%) was achieved using 1.5 × MS supplemented with 1.5 mg/L 6-BA, 0.2 mg/L ZT and 0.1 mg/L NAA (induction medium 7: ECM7). On MS medium, both lower (1.0 mg/L) and higher (2.0 mg/L) concentrations of 6-BA resulted in significantly reduced embryogenic callus formation (5.80% and 5.41%, respectively), demonstrating a dose-dependent response to cytokinin. These results indicate that embryogenic callus induction from YGC exhibits high sensitivity to both basal medium composition and cytokinin-auxin balance, highlighting the critical role of growth regulator optimization for effective induction.

### 2.6. Effects of Plant Growth Regulator Combinations for Morphological Maintenance of Embryogenic Callus During Subculture

Following the optimization embryogenic callus induction conditions from yellow-green compact callus (Table 3), this study examined the influence of PGR combinations on the morphological maintenance and embryogenic potential of embryogenic callus during subculture.

**Figure 6:**
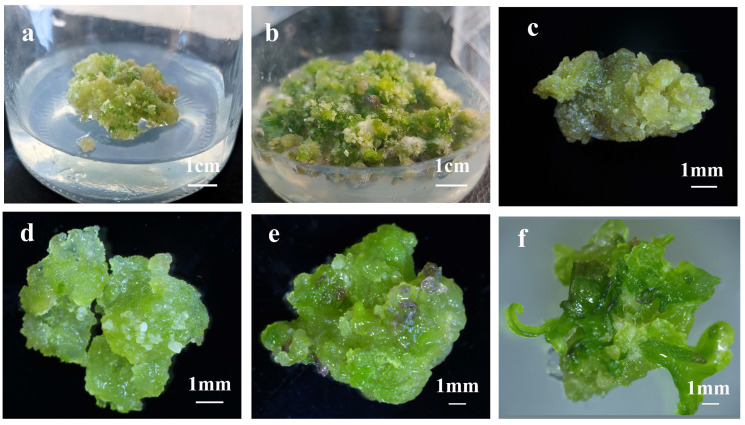
**Morphological response of embryogenic callus to varying concentrations of plant growth regulators during early subculture in *Catalpa bungei* (genotype 12–13).** Calli were cultured on MS basal medium supplemented with different combinations of 6-BA, ZT, and NAA for 20 days, with 3 biological replicates per treatment. (**a**) Initial granular embryogenic callus at the start of subculture (scale bar = 1 cm). (**b**) Proliferated embryogenic callus (EC) after 20 days of subculture, showing increased biomass (scale bar = 1 cm). (**c**) Non-embryogenic callus formed in the absence of ZT, with loose texture (scale bar = 1 mm). (**d**,**e**) Yellow-green, granular embryogenic callus maintained under optimal concentrations of 6-BA and ZT, retaining high embryogenic potential (scale bar = 1 mm for both). (**f**) Green and hard callus with abnormal shoot formation, indicating loss of embryogenic potential (scale bar = 1 mm).

The analysis revealed that while proliferation rates of embryogenic callus remained consistent across different PGR treatments, significant variations emerged in the morphological quality and developmental potential of the resulting calli (Figure 6a,b and Table 4). Under identical concentrations of 6-BA and NAA, the absence of ZT led to yellowish-brown, soft calli that lost their characteristic granular structure and embryogenic potential (Figure 6c; proliferation medium 1: EPM1). Conversely, the addition of ZT (0.2 mg/L) maintained embryogenic competence, as demonstrated by the formation of yellow-green, soft calli (Figure 6d; EPM2).

The concentration of 6-BA significantly influenced embryogenic callus morphology. Lower concentrations (0.6–0.8 mg/L) preserved the granular phenotype, with optimal results at 0.8 mg/L producing green and granular in callus (Figure 6e; EPM3)—characteristic of high embryogenic potential. However, higher 6-BA concentrations (1.2–1.5 mg/L) induced excessive shoot primordia formation, resulting in green, compact, and hard calli with abnormal shoot-like structures incapable of normal plantlet regeneration (Figure 6f; EMP4-EPM5).

Collectively, these results indicate that maintaining moderate cytokinin levels, particularly 0.8 mg/L 6-BA combined with 0.2 mg/L ZT, is essential for preserving the embryogenic identity and proliferation capacity of embryogenic callus during early subculture.

### 2.7. Effect of Plant Growth Regulator Combinations on Shoot Organogenesis

Following the establishment of embryogenic callus in *C. bungei* (Table 4), the study investigated shoot organogenesis as an alternative regeneration pathway. This approach generates adventitious shoots from embryogenic tissues and offers advantages in rapid regeneration speed, making it particularly valuable for large-scale propagation and genetic transformation applications.

To direct embryogenic callus toward shoot organogenesis, the study examined various PGR, specifically cytokinins (6-BA, KT, ZT, TDZ) and auxin (NAA), for their effects on shoot induction and bud formation (Table 5). We found that increasing 6-BA concentration from 0.3 to 0.6 mg/L significantly enhanced shoot induction. These hormones serve essential and often synergistic functions in shoot morphogenesis-cytokinin facilitating cell division and shoot initiation, while auxin regulates cell differentiation and tissue patterning [31,32,33,34]. Multiple combinations were evaluated to determine their impact on organogenic efficiency and adventitious bud formation per callus.

Among the cytokinins tested, increasing 6-BA concentration from 0 to 0.6 mg/L significantly enhanced shoot induction, with 0.6 mg/L achieving 85.64% induction rate (SOM3), substantially higher than 0 mg/L (SOM1) or 0.3 mg/L (SOM2) (*p* < 0.05). The combination of 0.6 mg/L 6-BA, 0.4 mg/L KT with 0.15 mg/L NAA further enhanced shoot organogenesis to 88.89% and yielded the highest number of adventitious buds (4.07 per callus) (SOM4).

Using identical levels of 6-BA and NAA, the addition of KT, ZT, and TDZ supported high shoot organogenesis rates and adventitious bud formation (SOM5-7) without significant different among these treatments (*p* > 0.05).

Regarding auxin, increasing NAA concentration from 0.02 to 0.15 mg/L significantly enhanced both shoot induction rates and bud proliferation, confirming its synergistic interaction with cytokinins.

In summary, shoot organogenesis in *C. bungei* was enhanced through optimization of PGR combinations, specifically with 0.6 mg/L 6-BA and 0.4 mg/L KT in combination with 0.15 mg/L NAA, establishing an efficient method for regeneration of *C. bungei*.

### 2.8. Additive Effects on Somatic Embryogenesis

Beyond evaluating PGRs for shoot organogenesis induction, this study examined somatic embryogenesis—a pathway that facilitates bipolar embryo development and typically yields higher genetic fidelity and whole-plant regeneration.

However, maintaining embryogenic callus alone does not ensure successful somatic embryo formation. Previous research has demonstrated that specific additives, such as ABA, PEG4000, and Phytagel, can influence embryo differentiation by regulating water availability, osmotic potential, and stress responses-factors critical to embryogenesis [28,35,36]. Consequently, this study evaluated these additives’ effects at various concentrations on somatic embryo induction in *C. bungei* (Table 6). All three additives markedly influenced somatic embryogenesis efficiency in a concentration-dependent manner. Among these, 5.0 g/L Phytagel achieved the highest somatic embryogenesis induction rate of 76.31% (somatic embryogenesis medium 8: SEM8), significantly exceeding that of 3.0 g/L Phytagel (SEM7) (*p* < 0.05). For ABA, the minimum tested concentration (0.0005 g/L) yielded the highest induction rate (67.22%, SEM1), while higher concentrations proved less effective (SEM2 and SEM3). PEG4000 showed a dose-dependent increase, reaching 64.99% at 10.0 g/L (SEM6).

Notably, no statistically significant differences merged among the optimal concentrations of the three additives, indicating that concentration exerts a greater influence than additive type on somatic embryogenesis induction in *C. bungei*.

### 2.9. Plantlet Regeneration from Somatic Embryos

**Figure 7:**
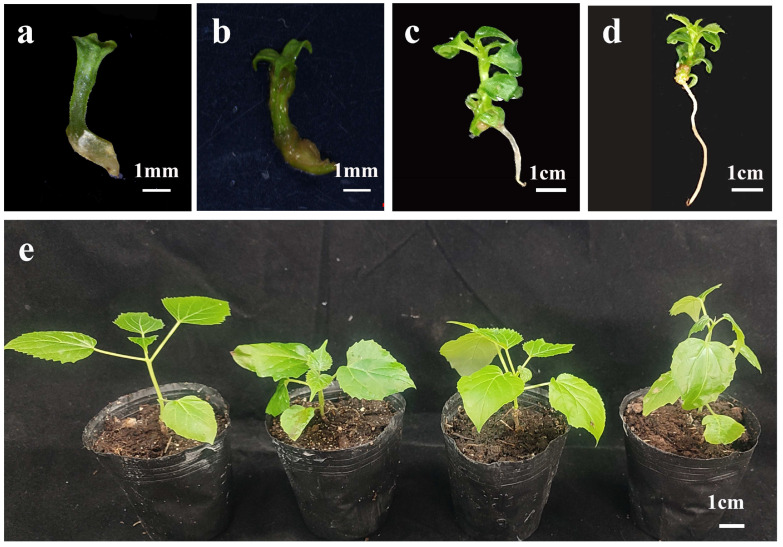
**Regeneration of whole plants from cotyledonary somatic embryos of *Catalpa bungei* (genotype 12–13).** When the cotyledons turned green, the somatic embryos were transferred to hormone-free 1/2 MS medium. (**a**,**b**) Cotyledonary somatic embryos initiating greening and cotyledon expansion, with radicle elongation (scale bar = 1 mm). (**c**) Development of multiple true leaves from the embryo axis (scale bar = 1 cm). (**d**) Complete plantlet with well-developed shoots and elongated root system (scale bar = 1 mm). After 30–40 days of culture, the embryonic roots elongated to 5–7 cm, and the plantlets were transplanted into nutrient pots containing peat soil, as shown by (**e**) successfully acclimatized plantlets after transplantation (scale bar = 1 cm).

To further evaluate the regenerative potential of somatic embryos in *C. bungei*, this study monitored their post-embryogenic development in vitro and following transplantation using cotyledon-derived somatic embryos as a representative example. The cotyledonary embryos initially displayed cotyledon expansion, greening, and radicle elongation (Figure 7a,b). Multiple true leaves subsequently developed from the shoot apex (Figure 7c), and the embryos matured into complete plantlets with well-defined shoot and root systems (Figure 7d). These plantlets were subsequently transferred to soil and successfully acclimatized, as evidenced by robust growth under ex vitro conditions (Figure 7e). This demonstrates that somatic embryos in *C. bungei* can complete the entire regeneration cycle and develop into viable whole plants.

## 3. Discussion

**Figure 8:**
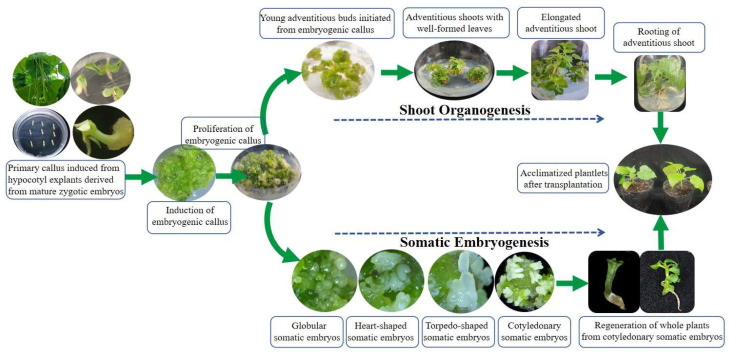
**Schematic diagram illustrating the two complete in vitro regeneration pathways (somatic embryogenesis and shoot organogenesis) of *C. bungei* (genotype 12–13) from embryogenic callus to rooted vitroplants.**

This study demonstrates that embryogenic callus in *C. bungei* possesses bipotency, capable of differentiating into either somatic embryos or adventitious shoots (Figure 8). This dual differentiation capacity expands upon previous findings in *C. bungei*, which primarily addressed unipolar regeneration. While similar plasticity has been documented in other woody species like *Paulownia tomentosa, Euryodendron excelsum, Cocos nucifera* L., *Paramignya trimera*, etc. [28,37,38], this research represents the first to systematic confirmation of this phenomenon in *C. bungei*, presenting important implications for both mass propagation and genetic transformation.

The heterogeneous characteristics of embryogenic callus likely indicate an early primed cellular state, where developmental pathways uncommitted, enabling cells to respond to external stimuli such as PGRs, basal medium or osmotic additives. This study established that cytokinin-rich conditions promote shoot organogenesis (achieving up to 88.89% efficiency with a combination of 6-BA, KT, and NAA), while stress-related additives including ABA, PEG4000, and Phytagel enhance somatic embryogenesis (reaching 76.31% efficiency). This developmental plasticity facilitates flexible regeneration strategies, allowing researchers to optimize culture conditions for either clonal propagation via shoots or embryogenic regeneration for genetic transformation, based on specific application requirements.

The successful induction of embryogenic callus is fundamental for plant regeneration, and research demonstrates its significant dependence on PGR and basal medium composition. Notably, explants derived from mature zygotic embryos of *C. bungei* showed no response to 2,4-D, yielding only brown, non-embryogenic callus. This observation differs from previous successful studies in *C. bungei* using immature embryos [30], and corresponds with findings in *Picea abies* and *Brassica napus* [39,40], where immature explants exhibit demonstrate enhanced auxin sensitivity due to elevated metabolic activity and increased expression of auxin-related genes (e.g., *ARF*, *PIN*). Alternatively, elevated concentrations of cytokinins, specifically 6-BA and ZT, effectively induced compact, YGC from mature explants. These YGC progressively developed into embryogenic callus when subcultured on media with decreased cytokinin levels. This sequential hormonal approach mirrors successful protocols in *Citrus sinensis* and *Carica papaya*, where initial high cytokinin concentrations promote dedifferentiation, and subsequent induction enables organized embryonic development [41,42].

Basal medium composition plays a pivotal role in regulating somatic embryogenesis and subsequent organ development of *C. bungei* genotype 12–13. DKW medium was effectively supportive of primary callus formation, whereas 1.5 × MS medium significantly enhanced EC induction—likely attributed to its elevated macro- and micronutrient concentrations, which better meet the high metabolic demands associated with EC initiation. This observation aligns with findings in *Canna* × *generalis*, where full-strength MS medium achieved an EC induction efficiency of 47.45% [43]. Notably, this medium-specific response exhibits genotype dependence. While other *C. bungei* genotypes have been shown to exhibit successful culture on DKW medium [29], genotype 12–13 displayed a clear preference for MS-based formulations during EC induction. This pattern mirrors the distinct medium responsiveness observed among *Eucalyptus dunnii* clones [44], a phenomenon commonly reported in woody plant tissue culture systems. For adventitious bud differentiation from ECs, DKW medium showed superior performance in our study, consistent with evidence from *Cedrela odorata* and *Cercis canadensis* where DKW medium supported adventitious shoot development [45,46]. Hormone-free 1/2MS medium alleviated osmotic stress, thereby facilitating root development. This result is in agreement with reports on *Camellia oleifera* (63.67% regeneration rate) [47], the optimal rooting of *Taiwania cryptomerioides* [48], and the species-specific requirement for low-salt conditions in *C. japonica* [49]. Collectively, the sequential medium strategy is tailored to address stage-specific nutritional and osmotic demands.

Histological analysis confirmed that *C. bungei* somatic embryos originate from single epidermal or subepidermal cells undergoing symmetric divisions, following canonical stages (globular, heart, torpedo, cotyledonary). This unicellular origin aligns with observations in *Arabidopsis thaliana*, *Medicago truncatula*, and other angiosperms [50,51], indicating a conserved morphogenetic pathway. The developmental timeline and structural characteristics showed substantial consistency with other woody species like *Quercus* and *Pinus*, emphasizing the broader applicability of this methodology.

While our results demonstrate distinct morphogenic responses of *C. bungei* tissues to varying PGRs, media, and additives—including differences in callus morphology, somatic embryo induction, shoot organogenesis, and adventitious bud formation—the underlying molecular mechanisms remain unclear. Future research should examine expression profiles of key developmental regulators-such as *WUSCHEL* (*WUS*), *LEAFY COTYLEDON1* (*LEC1*), and *AUXIN RESPONSE FACTORS* (*ARFs*)-using qRT-PCR or transcriptome sequencing. Similar molecular approaches have revealed stage-specific gene expression dynamics during somatic embryos in *Medicago truncatula*, *Pinus radiata*, *Coffea arabica* and *Tilia amurensis* Rupr [52,53,54,55]. Understanding these gene networks in *C. bungei* will facilitate the refinement of high-efficiency regeneration systems and inform molecular breeding strategies for this economically important tree

## 4. Materials and Methods

### 4.1. Plant Material and Explants Preparation

Fruits were collected from the half-sib family of *Catalpa bungei* (genotype 12–13) 80 days after full bloom, from the germplasm nursery in Nanyang City, Henan Province, China. *C. bungei* (genotype 12–13) is a hybrid progeny from the Luoqiu series, noted for its fast growth and favorable regeneration potential. The fruits were surface sterilized by scrubbing three times with cotton balls soaked in 75% ethanol. Seeds were extracted under sterile conditions and further sterilized by immersion in 75% ethanol for 1 min, followed by treatment with 2% (*v*/*v*) sodium hypochlorite for 15 min, and rinsed six times with sterile distilled water. Seed coats were removed, and mature zygotic embryos were aseptically excised for in vitro culture.

### 4.2. Primary Callus Induction

Zygotic embryos were initially cultured on CIM (9 explants/plate, 27 explants/replicate, 3 replicates). Once the cotyledons, hypocotyls, and plumules emerged, each part was cultured independently on CIM. CIM consisted of MS basal salts (Duchefa Biochemie, M0221, Haarlem, The Netherlands) supplemented with varying concentrations of 6-BA (0.5–2.0 mg/L), NAA (0.2–0.8 mg/L), and 2,4-D (0.5–2.0 mg/L) (Duchefa Biochemie, M0221, The Netherlands) (Table 1). Media were solidified with 0.3% (*w*/*v*) Phytagel (Sigma-Aldrich, P8169, St. Louis, MO, USA) and contained 3% (*w*/*v*) sucrose, with pH adjusted to 5.6–5.8 prior to autoclaving at 121 °C for 20 min. Cultures were maintained in darkness at 25 ± 1 °C and subcultured every 3 weeks.

### 4.3. Subculture of Primary Callus

Green and compact primary calli from cotyledons, hypocotyls, and plumules were transferred to SCM (9 calli/plate, 18 calli/replicate, 3 replicates). SCM was based on DKW (Duchefa Biochemie, D0650, Haarlem, The Netherlands), WPM (Duchefa Biochemie, W0100, Haarlem, The Netherlands) or MS basal media supplemented with 6-BA (0.5–2.0 mg/L), NAA (0.01–0.5 mg/L), and ZT (Sigma-Aldrich, Z0278, St. Louis, MO, USA) (0.2, 0.5, and 1.0 mg/L) (Table 2). Cultures were incubated under a 16 h light/8 h dark photoperiod with 50 µmol m^−2^ s^−1^ light intensity provided by 70% red and 30% blue LEDs [56,57] at 25 ± 1 °C.

### 4.4. Embryogenic Callus Induction and Proliferation

Compact, yellow-green calli were selected and cultured on embryogenic callus ECM (9 calli/plate, 18 calli/replicate, 3 replicates), based on DKW, MS, or 1.5 × MS salts, supplemented with 6-BA (1.0–2.0 mg/L), ZT (0.2 mg/L), and either NAA or IBA (0.1 mg/L) (Table 3). For proliferation, embryogenic calli were subcultured on EPM, consisting of MS basal salts with 6-BA (0.6–1.5 mg/L), NAA (0.1 mg/L), and ZT (0.2 mg/L) (Table 4).

### 4.5. Somatic Embryogenesis and Shoot Organogenesis

Embryogenic calli were cultured on either SEM or SOM (20 calli/plate, 3 replicates). SEM was composed of 1/2 MS medium (Duchefa Biochemie, M0221, Haarlem, The Netherlands) supplemented with ABA (Sigma-Aldrich, A1049, St. Louis, MO, USA) (0.005, 0.002, and 0.005 g/L), PEG4000 (Sigma-Aldrich, P3640, St. Louis, MO, USA) (2.0, 5.0, and 10.0 g/L) and phytagel (3.0, 5.0, and 8.0 g/L) (Table 5). When the cotyledons turned green, the somatic embryos were transferred to hormone-free 1/2 MS medium, where they developed into complete plantlets. After 30–40 days, the embryonic roots elongated to 5–7 cm and the plantlets were transplanted into nutrient pots containing peat soil.

SOM contained DKW basal medium and various cytokinins including 6-BA, ZT, TDZ, and KT (Table 6). When adventitious shoots reached 3–5 cm, they were transferred to rooting medium (DKW + 0.15 mg/L NAA + 1.5 mg/L IBA).

### 4.6. Histological Analysis

Samples were collected at key stages of somatic embryogenesis and shoot organogenesis and fixed in FAA solution (Duchefa Biochemie, F0502, Haarlem, The Netherlands) (formaldehyde:acetic acid:70% ethanol = 1:1:18) for one week. Samples were dehydrated in graded ethanol, cleared with xylene, and embedded in paraffin. Sections (8–10 µm thick) were cut using a rotary microtome (LEICA RM2245), stained with hematoxylin, and visualized under a light microscope (LEICA ICC50 HD, Wetzlar, Germany).

### 4.7. Statistical Analysis

Callus induction rates were recorded at 20 days. Embryogenic callus was identified by yellow-green granules or visible somatic embryos and evaluated after two subcultures (40–45 days). Somatic embryogenesis induction was defined as the presence of ≥5 white embryos per callus piece and evaluated after 30 days. Shoot organogenesis rates were calculated based on the number of adventitious shoots.

Data were analyzed using Analysis of Variance (ANOVA), and Post hoc multiple comparisons were performed using Duncan’s Multiple Range Test (DMRT) at a significance level of *p* ≤ 0.05 with OriginLab Corporation, Northampton, MA, USA.

## 5. Conclusions

This research established two in vitro propagation systems encompassing somatic embryogenesis and shoot organogenesis in *C. bungei* (12–13). Histological analysis documented the initiation and development of somatic embryos. The application of cytokinin (6-BA and ZT) demonstrates efficiency for embryogenic callus induction using mature seed embryos as explants. Elevated concentrations of 6-BA 2.0 mg/L and ZT 1.0 mg/L serve as critical factors in inducing yellow-green and compact callus, representing an essential transition from primary callus to embryogenic callus. The basal media (DKW and MS) are crucial factors in modulating callus state and inducing embryogenic callus. The combination of 6-BA and KT effectively enhances adventitious bud differentiation. Phytagel (5.0 g/L) in the culture medium facilitates somatic embryo development and maturation. These findings contribute valuable insights for the propagation and transgenic breeding of *C. bungei*.

## Figures and Tables

**Table 1:** Effect of different concentrations and combinations of plant growth regulators on primary callus induction of *Catalpa bungei* (genotype 12–13).

Treatment	Cytokinin	Auxin		Yellow Callus Induction Rate %	Green Callus Induction Rate %	Brown Callus Induction Rate %
	6-BA (mg/L)	NAA (mg/L)	2,4-D (mg/L)			
CIM1	0.5	0.2	0	29.96 ± 7.76 c	46.63 ± 10.42 b	3.00 ± 0.29 e
CIM2	1.0	0.2	0	41.88 ± 17.79 b	43.16 ± 12.11 bc	9.62 ± 2.26 c
CIM3	1.5	0.2	0	31.86 ± 9.70 c	57.04 ± 18.48 a	6.54 ± 1.90 d
CIM4	2.0	0.2	0	18.64 ± 6.81 d	58.30 ± 11.05 a	20.33 ± 5.22 b
CIM5	1.0	0.4	0	19.23 ± 0.31 d	55.14 ± 0.14 a	17.56 ± 0.16 b
CIM6	2.0	0.8	0	59.94 ± 13.90 a	35.09 ± 7.25 c	4.66 ± 1.91 e
CIM7	0.1	0	0.5	0	0	48.50 ± 4.25 a
CIM8	0.1	0	1.0	0	0	9.85 ± 1.30 c
CIM9	0.1	0	2.0	0	0	7.95 ± 0.50 cd

CIM = callus induction medium. Each treatment was conducted with 3 biological replicates, and values are presented as mean ± standard deviation (SD). Different letters within a column indicate statistically significant differences according to Duncan’s multiple range test (*p* ≤ 0.05). Note: Values of “0” in yellow callus % and green callus % for CIM7–CIM9 indicate that no yellow or green callus was induced under these treatments.

**Table 2:** Effect of basal medium and plant growth regulators concentrations during subculture on the induction of yellow-green compact callus in *C. bungei* (genotype 12–13).

Treatment	Basal Medium	Cytokinin		Auxin	Yellow-Green Callus Induction Rate %
		6-BA (mg/L)	ZT (mg/L)	NAA (mg/L)	
SCM1	DKW	0.5	0.2	0.1	37.53 ± 5.59 ab
SCM2	DKW	1.0	0.2	0.1	26.89 ± 9.33 c
SCM3	DKW	2.0	0.2	0.1	26.58 ± 3.78 c
SCM4	DKW	2.0	0.5	0.1	35.09 ± 2.64 b
SCM5	DKW	2.0	1.0	0.1	45.73 ± 6.17 a
SCM6	DKW	1.0	0.2	0.01	7.69 ± 2.51 e
SCM7	DKW	1.0	0.2	0.05	24.50 ± 1.93 cd
SCM8	WPM	1.0	0.2	0.1	16.92 ± 5.36 d
SCM9	WPM	2.0	1.0	0.1	20.73 ± 7.01 d
SCM10	MS	0.5	0.2	0.1	3.09 ± 2.01 f
SCM11	MS	1.0	0.2	0.1	8.87 ± 2.23 e

SCM = subculture medium. Each treatment was performed with 3 biological replicates, and values are presented as mean ± standard deviation (SD). Different letters within a column indicate statistically significant differences according to Duncan’s multiple range test (*p* ≤ 0.05).

**Table 3:** Effect of different basal medium and combinations of plant growth regulators on embryogenic callus induction from subcultured yellow-green compact callus in *C. bungei* (genotype 12–13). The embryogenic callus (EC) induction rate (%) was recorded after 20 days of culture.

Treatment	Basal Medium	Cytokinin		Auxin	Embryogenic Callus Induction Rate %
		6-BA (mg/L)	ZT (mg/L)	NAA (mg/L)	
ECM 1	DKW	1.5	0.2	0.1	0 c
ECM 2	DKW	2.0	0.2	0.1	0 c
ECM 3	DKW	2.0	0.2	0	0 c
ECM 4	MS	1.0	0.2	0.1	5.41 ± 0.67 b
ECM 5	MS	1.5	0.2	0.1	12.67 ± 2.45 a
ECM 6	MS	2.0	0.2	0.1	5.80 ± 1.05 b
ECM 7	1.5 × MS	1.5	0.2	0.1	16.57 ± 3.02 a

ECM = embryogenic callus induction medium. Each treatment was performed with 3 biological replicates, and values are presented as mean ± standard deviation (SD). Different letters within a column indicate statistically significant differences according to Duncan’s multiple range test (*p* ≤ 0.05).

**Table 4:** Effects of plant growth regulators on the morphological maintenance of embryogenic callus derived from yellow-green and compact in *C. bungei* (genotype 12–13). Calli were assessed after 20 days of subculture for color, texture, and abnormal structure formation to evaluate callus status.

Treatment	Cytokinin		Auxin	Status of Embryogenic Callus
	ZT (mg/L)	6-BA (mg/L)	NAA (mg/L)	
EPM1	0	0.6	0.1	Yellowish-brown, soft
EPM2	0.2	0.6	0.1	Yellow-green, soft
EPM3	0.2	0.8	0.1	Green, granular
EPM4	0.2	1.2	0.1	Green callus, abnormal shoot
EPM5	0.2	1.5	0.1	Green callus, abnormal shoot

EPM = embryogenic callus maintenance medium. Each treatment was conducted with 3 biological replicates to ensure result reliability.

**Table 5:** Effect of types and concentration of plant growth regulators on shoot organogenesis and adventitious bud formation in *C. bungei* (genotype 12–13).

Treatment	Cytokinin				Auxin	Shoot Organogenesis Rate %	Number of Adventitious Buds per Callus
	6-BA (mg/L)	KT (mg/L)	ZT (mg/L)	TDZ (mg/L)	NAA (mg/L)		
SOM1	0.0	0.4	0	0	0.02	63.59 ± 20.49 c	1.33 ± 0.24 c
SOM2	0.3	0.4	0	0	0.02	61.11 ± 35.00 c	2.80 ± 0.41 b
SOM3	0.6	0.4	0	0	0.02	85.64 ± 12.29 a	2.79 ± 0.28 b
SOM4	0.6	0.4	0	0	0.15	88.89 ± 15.71 a	4.07 ± 0.64 a
SOM5	0.6	0.2	0	0	0.15	78.09 ± 14.29 a	3.89 ± 0.43 ab
SOM6	0.6	0	0.2	0	0.15	72.29 ± 13.31 b	4.06 ± 0.57 a
SOM7	0.6	0	0	0.2	0.15	75.29 ± 13.35 b	4.05 ± 0.24 a

SOM = shoot organogenesis medium. Each treatment was conducted with 3 biological replicates, and values are presented as mean ± standard deviation (SD). Different letters within a column indicate statistically significant differences according to Duncan’s multiple range test (*p* ≤ 0.05).

**Table 6:** Effect of different additives and their concentrations on somatic embryogenesis induction in *C. bungei* (genotype 12–13).

Treatment	Additive	Concentration (g/L)	Somatic Embryogenesis Induction Rate %
SEM1	ABA	0.0005	67.22 ± 10.07 a
SEM2	ABA	0.002	40.34 ± 4.48 bcd
SEM3	ABA	0.005	28.58 ± 5.97 d
SEM4	PEG4000	2.0	37.50 ± 8.84 cd
SEM5	PEG4000	5.0	59.44 ± 8.58 bc
SEM6	PEG4000	10.0	64.99 ± 8.18 ab
SEM7	Phytagel	3.0	45.15 ± 4.72 bc
SEM8	Phytagel	5.0	76.31 ± 5.28 a
SEM9	Phytagel	8.0	66.07 ± 2.24 a

Phytagel = a plant-derived gelling agent; SEM = somatic embryogenesis medium. Each treatment was conducted with 3 biological replicates, and values are presented as mean ± standard deviation (SD). Different letters within a column indicate statistically significant differences according to Duncan’s multiple range test (*p* ≤ 0.05).

## Data Availability

All relevant data are within the manuscript.
